# Supplementary material for: The Translation of Mobile-Exoneuromusculoskeleton-Assisted Wrist–Hand Poststroke Telerehabilitation from Laboratory to Clinical Service
Source: Bioengineering (Basel). 2023 Aug 18;10(8):976. doi: 10.3390/bioengineering10080976 (PMC10451942; doi:10.3390/bioengineering10080976)
Supplement: Supplementary file 1 [file bioengineering-10-00976-s001.zip › bioengineering-2544110-supplementary.pdf]

Table S1. Usefulness, Satisfaction and Ease of Use (USE) Questionnaire. Scores on seven-point Likert scales (1 “strongly disagree” to 7 “strongly agree”)

**Usefulness**
It helps me be more effective
It helps me be more productive
It is useful
It gives me more control over my（therapeutic）activities
It makes the things I want to accomplish easier to get done
It saves me time when I use it
It meets my needs
It does everything I would expect it to do
**Ease of Use**
It is easy to use
It is simple to use
It is user friendly
It requires the fewest steps possible to accomplish what I want to do with it
It is flexible
Using it is effortless
I can use it without written instructions
I don’t notice any inconsistencies as I use it
Both occasional and regular users would like it
I can recover from mistakes quickly and easily
I can use it successfully every time
**Ease of Learning**
I learned to use it quickly
I easily remember how to use it
It is easy to learn to use it
I quickly became skillful with it
**Satisfaction**
I am satisfied with it
I would recommend it to a colleague
It is fun to use
It works the way I want it to work
It is wonderful
I feel I need to have it
It is pleasant to use

Table S2. Customized Intrinsic Motivation Inventory (IMI) used in the study.

Scores on seven-point Likert scales (1 “not at all true” to 7 “very true”). For an item with an (R) shown after, subtracted the item response from 8, and used the resulting number as the item score.

**Interest/Enjoyment**
I enjoyed doing this activity very much.
I thought this was a boring activity. (R)
This activity did not hold my attention at all. (R)
I thought this activity was quite enjoyable.
**Perceived Competence**
I think I am pretty good at this activity.
I am satisfied with my performance at this task.
I was pretty skilled at this activity.
This was an activity that I couldn’t do very well. (R)
**Effort/Importance**
I put a lot of effort into this.
I didn’t try very hard to do well at this activity. (R)
It was important to me to do well at this task.
I didn’t put much energy into this. (R)
**Pressure/Tension**
I felt very tense while doing this activity.
I was very relaxed in doing these. (R)
I was anxious while working on this task.
I felt pressured while doing these.
**Perceived Choice**
I felt like it was not my own choice to do this task. (R)
I felt like I had to do this. (R)
I did this activity because I had no choice. (R)
I did this activity because I wanted to.
**Value/Usefulness**
I believe this activity could be of some value to me.
I would be willing to do this again because it has some value to me.
I think doing this activity could help me to improve motor function of the affected upper limb.
I think this is an important activity.
**Relatedness**
I felt really distant to this person. (R)
I felt like I could really trust this person.
I’d like a chance to interact with this person more often.
It is likely that this person and I could become friends if we interacted a lot.
